# The Notch-mediated circuitry in the evolution and generation of new cell lineages: the tooth model

**DOI:** 10.1007/s00018-023-04831-7

**Published:** 2023-06-18

**Authors:** Thimios A. Mitsiadis, Pierfrancesco Pagella, Terence D. Capellini, Moya Meredith Smith

**Affiliations:** 1 Institute of Oral Biology, Centre for Dental Medicine, University of Zurich, Plattenstrasse 11, 8032 Zurich, Switzerland; 2 Wallenberg Center for Molecular Medicine (WCMM) and Department of Biomedical and Clinical Sciences, Linköpings Universitet, 581 85 Linköping, Sweden; 3 Department of Human Evolutionary Biology, Harvard University, Cambridge, MA 02138 USA; 4 Centre for Craniofacial and Regenerative Biology, Faculty of Dentistry, King’s College London, London, UK

**Keywords:** Notch signaling, Tooth development, Enamel, Cell commitment, Enamel organ, Ameloblasts, Evolution, Jagged

## Abstract

The Notch pathway is an ancient, evolutionary conserved intercellular signaling mechanism that is involved in cell fate specification and proper embryonic development. The *Jagged2* gene, which encodes a ligand for the Notch family of receptors, is expressed from the earliest stages of odontogenesis in epithelial cells that will later generate the enamel-producing ameloblasts. Homozygous Jagged2 mutant mice exhibit abnormal tooth morphology and impaired enamel deposition. Enamel composition and structure in mammals are tightly linked to the enamel organ that represents an evolutionary unit formed by distinct dental epithelial cell types. The physical cooperativity between Notch ligands and receptors suggests that *Jagged2* deletion could alter the expression profile of Notch receptors, thus modifying the whole Notch signaling cascade in cells within the enamel organ. Indeed, both *Notch1* and *Notch2* expression are severely disturbed in the enamel organ of *Jagged2* mutant teeth. It appears that the deregulation of the Notch signaling cascade reverts the evolutionary path generating dental structures more reminiscent of the enameloid of fishes rather than of mammalian enamel. Loss of interactions between Notch and Jagged proteins may initiate the suppression of complementary dental epithelial cell fates acquired during evolution. We propose that the increased number of Notch homologues in metazoa enabled incipient sister cell types to form and maintain distinctive cell fates within organs and tissues along evolution.

## Introduction

The Notch pathway is an evolutionarily conserved signaling mechanism that enables adjacent cells to adopt different fates [1–5]. In *Drosophila*, the *Notch* gene encodes a transmembrane receptor with a large extracellular domain carrying multiple epidermal growth factor (EGF)-like repeats and a cytoplasmic domain required for signal transduction. The Notch receptor interacts with membrane-bound ligands encoded by the *Delta* and *Serrate* genes that in their extracellular domain contain the DSL domain (Delta, Serrate, Lag-2). The DSL domain is required for interaction of ligands with the Notch receptor [6, 7]

In vertebrates, four genes encoding Notch receptors (*Notch1*, *Notch2, Notch3*, and *Notch4*) and five genes encoding ligands for the Notch receptors (*Jagged1, Jagged2*, *Delta-like1*, *Delta-like3*, and *Delta-like4*) have been identified [5, 8, 9]. The signal induced by ligand binding is transmitted at the intracellular part of the receptor in a process involving proteolysis and interactions with cytoplasmic and nuclear proteins [1, 10–17]. Signals exchanged between neighboring cells through the Notch receptors influence cell fate determination, differentiation, proliferation and apoptotic events at all stages of development, thus controlling organ formation and morphogenesis [8, 9, 17–20]. The increasing number of Notch homologues in vertebrates, together with the absence of *Notch* genes in non-metazoans, suggests a role for the Notch signaling pathway in the establishment of complex body plans [21, 22]. Notch malfunction has been shown to disrupt aspects of neurogenesis, somite formation, angiogenesis, kidney and lymphoid development [9, 16, 23–30]. In humans, mutations in the *Notch1*, *Notch3* and *Jagged1* genes are associated, respectively, with a neoplasia (a T-cell acute lymphoblastic leukemia/lymphoma), a late onset neurological disease known as CADASIL (cerebral autosomal dominant arteriopathy with subcortical infarcts and leukoencephalopathy) and a complex inherited disorder known as Alagille syndrome (affecting mainly the liver, heart, vertebrae, eye and face) [9, 31–34].

Several studies have demonstrated that Notch signaling is involved in tooth morphology and enamel matrix deposition [35–42]. Teeth are organs that arise from progressive reciprocal inductive interactions between the stomodeum epithelium and the underlying neural crest-derived mesenchyme that transform the tooth primordia into complex mineralized structures with various cell types, among which the epithelial-derived ameloblasts that synthesize and secrete the organic components of the enamel [43–45]. Initiation of odontogenesis is visualized as local epithelial thickenings of the oral epithelium, at the sites of the future teeth [45–47]. Thereafter, the thickened epithelium grows and forms the dental bud and cap structures that mark the onset of the tooth morphology. Subsequent epithelial folding and growth gives rise to a bell structure where cytodifferentiation events start. In mammals, four clearly distinct dental epithelial cell layers (i.e., inner enamel epithelium, stratum intermedium, stellate reticulum and outer enamel epithelium) are present at this stage that are important for amelogenesis. However, to date, there is not much information concerning the role of the Notch signaling pathway in gradual cell fate determination and differentiation of these dental epithelial cell lineages.

Since the core Notch pathway requires two adjacent cells in direct contact with each other, we examined if *Jagged2* deletion affects *Notch1* and *Notch2* expression in the epithelium of developing mouse teeth. We have recently shown that epithelial deletion of *Jagged1* deregulated the expression of both *Notch1* and *Notch2* in dental epithelium [48]. Based on our recent findings and the results obtained here we suggest a hypothetical model involving molecules of the Notch signaling pathway in dental epithelial cell morphotype and function. The proposed model could unravel a general, functional correlation between the evolution of the discrete expression of Notch receptors and its ligands and the evolution of specialized dental cell types.

## Materials and methods

### Notch receptors and ligands in evolution

To obtain an overview of the Notch receptors and ligands in evolution and their conservation among vertebrates, we screened available protein sequences from representative species of five main classes: fishes (Zebrafish—*Danio rerio*), amphibians (Western Clawed Frog—*Xenopus tropicalis*), reptiles (Common Wall Lizard—*Podarcis muralis*), birds (Chicken—*Gallus gallus*), and mammalians (Human—*Homo sapiens*). We then aligned all receptors and ligands sequences via ClustalW [49].

### Animals and tissue preparation

All mice (C57Bl/6) were maintained and handled according to the Swiss Animal Welfare Law and in compliance with the regulations of the Cantonal Veterinary office, Zurich (License 11/2014). Mouse embryos from embryonic day 12.5 (E12.5) to E18.5 were used for in situ hybridization. *Jag2*^*DDSL*^ mutant mice have been described previously [50, 51]. E12.5–E18.5 wild type, *Jagged2*^*+*/−^ and *Jagged2*^*−/−*^ mouse embryos were obtained by intercrossing *Jag2*^*DDSL/*+^ mice. Embryonic age was determined according to the appearance of the vaginal plug (day 0) and confirmed by morphological criteria. Pregnant females were sacrificed by cervical dislocation and the embryos were removed in Dulbecco’s phosphate-buffered saline (PBS). Dissected heads were fixed in 4% paraformaldehyde (PFA) for 24 h at 4 °C and prepared for sectioning.

### Probes and in situ hybridization

Digoxigenin- and fluorescein-labeled (Boehringer Manhnheim) antisense riboprobes for *Jagged2*, *Notch1* and *Notch2* were used [40, 51]. In situ hybridization on cryosections of E12.5–E18.5 embryos were performed as previously described [39, 40, 52]. Double in situ was performed using first the fluorescein probe, followed by the digoxigenin one.

## Results

### Overview of Notch receptors and ligands in vertebrates

**Fig. 1:**
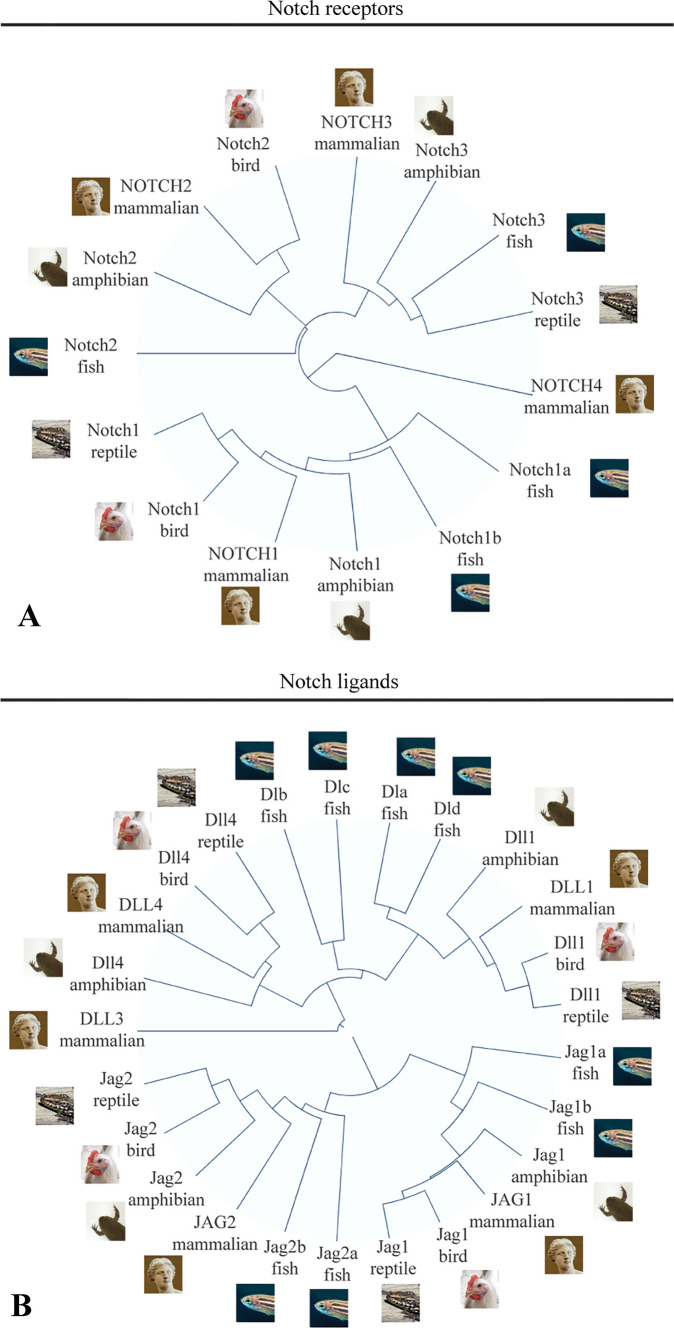
Overview of Notch receptors and ligands among vertebrates. **A** Circular phylogram (guide tree) obtained from the alignment of protein sequences (ClustalW) of Notch receptors from five selected vertebrate species. **B** Circular phylogram (guide tree) obtained from the alignment of protein sequences (ClustalW) of Notch ligands from five selected vertebrate species. Notice that connections indicate sequence similarities as determined from multiple alignment (5 iterations) and do not necessarily imply phylogenetic relationships. Fish: *Danio rerio* (Zebrafish); Amphibian: Xenopus tropicalis (Western Clawed Frog); Reptile: Podarcis muralis (Common Wall Lizard); Bird: *Gallus gallus* (Chicken); Mammalian: *Homo sapiens* (Human)

Alignement of representative protein sequences of all Notch receptors and ligands in species of five main classes (fishes, amphibians, reptiles, birds, mammalians) showed that Notch1, Notch2 and Notch3 were broadly identified in these classes. Notch orthologues in different classes showed higher sequence identity than paralogues within the same class (Fig. 1A). Notch4 was identified only in mammalians, and represented a clear side branch. Notch ligands clustered separately in the families of Jagged and Delta-like (Dll) ligands (Fig. 1B). Similarly to the Notch receptors, Notch ligands showed higher identity between orthologues, with Jagged1, Jagged2, Delta-like1 (Dll1), Delta-like3 (Dll3), Delta-like4 (Dll4) forming each separate branches.

### Notch1, Notch2 and Jagged2 expression during embryonic tooth development

**Fig. 2:**
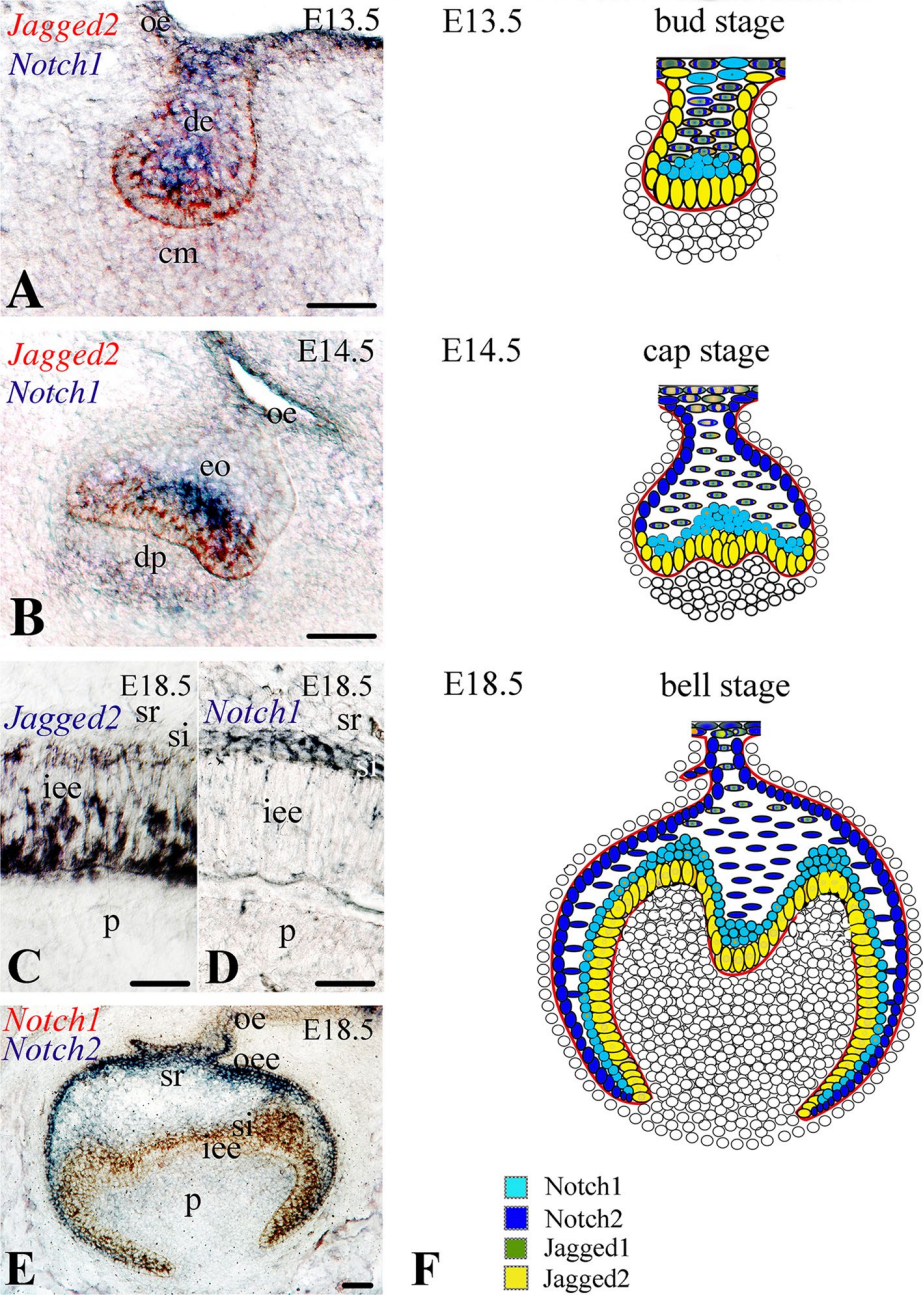
Comparative analysis of the expression patterns of *Jagged2, Notch1* and *Notch2* in dental epithelium during embryonic tooth development. In situ hybridization on frontal cryosections of E13.5–E18.5 mouse embryos (**A**–**E**). **A** At E13.5 (bud stage), *Jagged2* transcripts (red color) are detected in dental epithelial cells in contact with the condensed mesenchyme (cm), while *Notch1* mRNA (blue color) is observed in the neighboring to the *Jagged2*-expressing cells. **B** At E14.5 (cap stage), *Jagged2* transcripts (red color) are found in dental epithelial cells contacting the dental papilla mesenchyme (dp), whereas *Notch1* transcripts (blue color) are restricted to cells overlying the *Jagged2*-expressing cells. **C**, **D** At E18.5 (bell stage), *Jagged2* expression is restricted in cells of the inner enamel epithelium (iee) (**C**), while expression of *Notch1* is delimited to cells of the overlying stratum intermedium layer (si) (**D**). **E** At the bell stage (E18.5), *Notch1* transcripts (red color) are detected in cells of the stratum intermedium, while *Notch2* mRNA (blue color) is observed in cells of the stellate reticulum (sr). **F** Schematic representation of the expression patterns of Notch1, Notch2, Jagged1 and Jagged2 in dental epithelium of E13.5–E18.5 molar tooth germs. *de* dental epithelium, *oe* oral epithelium, *oee* outer enamel epithelium; *p* dental pulp. Scale bars **A**, **B**, **E** = 100 μm; **C**, **D** = 25 μm

**Fig. 3:**
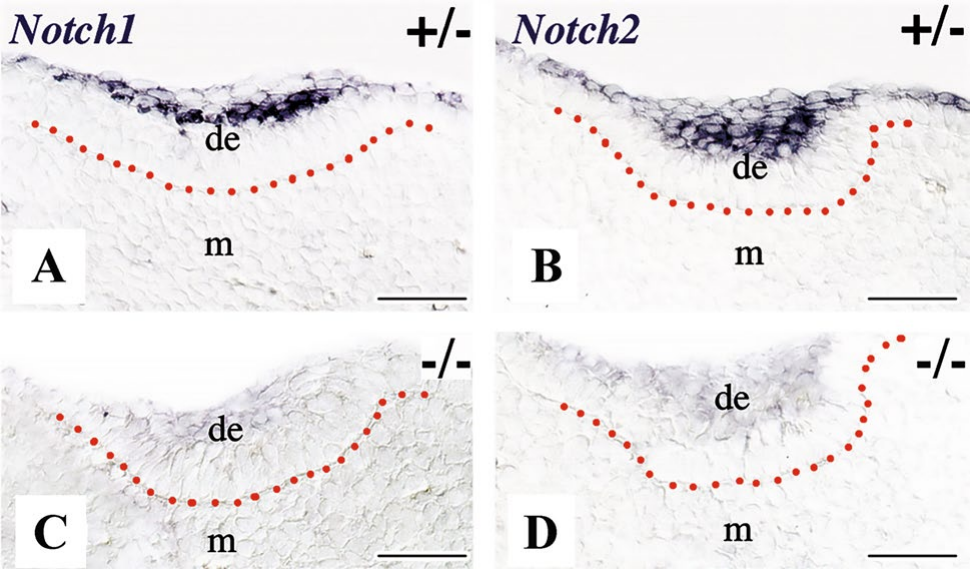
Expression of the *Notch1* and *Notch2* genes in the developing tooth areas of E12.5 *Jagged2* heterozygous (+/−) and homozygous (^***−/−***^) mouse embryos. Genotypes are indicated in each panel. In situ hybridization on frontal cryosections. Red dotted lines represent the borders between the dental epithelium (de) and mesenchyme (m). *Notch1* (**A**) and *Notch2* (**B**) are expressed in distinct cell populations of the dental epithelium in heterozygous embryos. *Notch1* (**C**) and *Notch2* (**D**) genes are not detected in dental tissues of homozygous embryos. Scale bars 100 μm

**Fig. 4:**
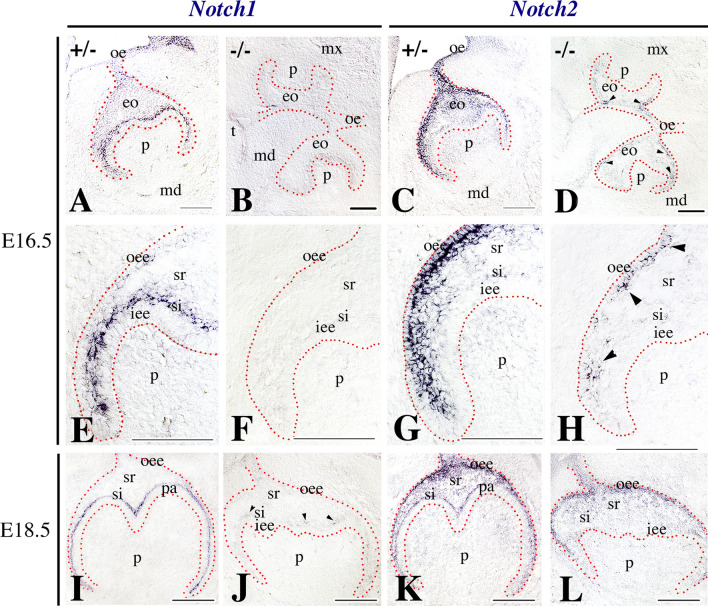
Comparison of the expression patterns of the *Notch1* and *Notch2* genes in developing teeth of E16.5 and E18.5 *Jagged2* heterozygous (+/−) and homozygous (***−/−***) mouse embryos. Genotypes are indicated in each panel. In situ hybridization on frontal cryosections. Red dotted lines represent the borders between the enamel organ (eo) and the surrounding mesenchyme. Arrowheads indicate *Notch1* or *Notch2* expressing cells in *Jagged2*^***−/−***^ embryos. Distinct expression patterns of *Notch1* (**A**, **E**, **I**) and *Notch2* (**C**, **G**, **K**) in the dental epithelium of E16.5 and E18.5 *Jagged2* ± embryos. Higher magnifications show that *Notch1* is expressed in stratum intermedium (si) (**E**), while *Notch2* is expressed in cells of the outer enamel epithelium (oee) and stellate reticulum (sr) (**G**). *Notch1* (**B**, **F**, **J**) and *Notch2* (**D**, **H**, **L**) are downregulated in the dental epithelium of E16.5 and E18.5 *Jagged2*^***−/−***^ embryos. Also, note the fusions between the maxillary (mx) and mandibular processes (md) (**B**, **D**) in homozygous embryos. Higher magnifications show that *Notch1* is not detected in stratum intermedium (**F**), while *Notch2* expression is greatly reduced in cells of the outer enamel epithelium and stellate reticulum (**H**) in dental tissues of E16.5 *Jagged2*^***−/−***^ embryos. *iee* inner enamel epithelium, *oe* oral epithelium, *p* dental papilla mesenchyme, *pa* preameloblasts, *t* tongue. Scale bars 200 μm

To be able to interpret the role of the Notch signaling pathway in tooth evolution linked to the cell diversity of the enamel organ we first determined the expression pattern of *Notch1*, *Notch2* and *Jagged2* in development from sections of E12.5–E18.5 mouse teeth. At E12.5–13.5 dental epithelium (bud stage), *Jagged2* expression was observed in epithelial cells in contact with the condensed mesenchyme (Fig. 2A, red color), while *Notch1* and *Notch2* transcripts were detected in distinct epithelial territories adjacent to the *Jagged2*-expressing cells (Fig. 2A, blue color; Fig. 3A, B). During the cap stage (E14.5–E15.5), *Jagged2* transcripts were detected in dental epithelial cells contacting the dental papilla mesenchyme (Fig. 2B, red color), whereas *Notch1* labeling was restricted to cells overlying the *Jagged2*-expressing cells (Fig. 2B, blue color). At the bell stage (E16.5–E18.5), *Jagged2* expression was observed in cells of the inner enamel epithelium (Fig. 2C), while expression of *Notch1* was found in a differentiated cell layer behind, as the stratum intermedium (Fig. 2D; Fig. 2E, red color; Fig. 4A, E, I) and that of *Notch2* in middle cells of the stellate reticulum and as well as in outer enamel epithelium (Fig. 2E, blue color; Fig. 4C, G, K).

### ***Downregulation of Notch1 and Notch2 expression in Jagged2***^***−/−***^*** teeth***

To analyze the effects of *Jagged2* deletion in other molecules of the Notch signaling pathway, we examined the expression of *Notch1 and Notch2* in teeth of E12.5–E18.5 *Jagged2* deficient embryos. At E12.5 (early bud stage), the expression of both *Notch1* (Fig. 3C) and *Notch2* (Fig. 3D) was severely downregulated in the dental epithelium of homozygous mutant embryos. Downregulation of these two genes, but to a lesser extent for *Notch2* when compared to *Notch1*, persisted at more advanced developmental stages. Very few, if not at all, *Notch1* transcripts were detected in cells of the stratum intermedium of E16.5 (Fig. 4B, F) and E18.5 (Fig. 4J) *Jagged2*^***−/−***^ mouse embryo teeth. Similarly, *Notch2* expression was greatly reduced in cells of the outer enamel epithelium and stellate reticulum of E16.5 (Fig. 4D, H) and E18.5 (Fig. 4L) *Jagged2*^***−/−***^ teeth.

## Discussion

**Fig. 5:**
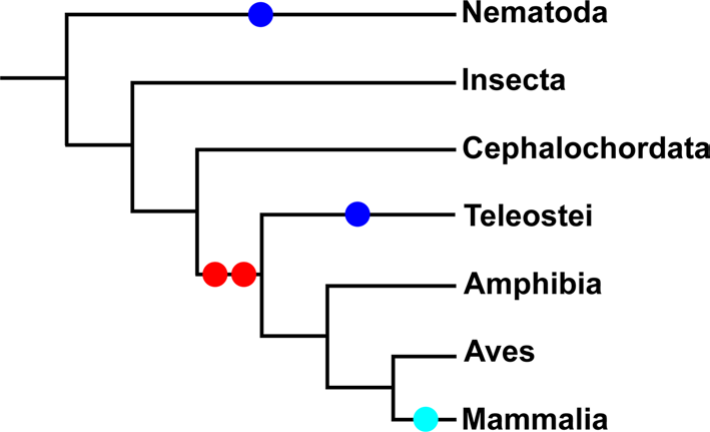
Evolutionary scenario of Notch duplication events. Commonly accepted tree of the taxa was extracted from NCBI taxonomy browser [21]. Spots indicate duplication events in the Notch family. Red spot: two duplication events prior to the differentiation of Teleostei and Tetrapoda. Dark blue spots: independent recent duplication events, one for Notch1 in Teleostei and one for Notch in nematode. Light blue: possible independent duplication event that gave rise to Notch4 in mammalian lineage. Alternatively, Notch4 could have been present already before the differentiation of Teleostei and Tetrapoda but lost along all lineages except Mammalia. Figure adapted from [21]

Evolutionary processes have contributed to the extensive diversification of cell types in animals. Cell homology in an increased number of new cell types that appeared during animal evolution could be due to inheritance from a common precursor [53, 54]. Notch signaling is an ancient, evolutionarily conserved signaling pathway that allows distinctive cell types with defined functions to be delineated and compared within and between species [1]. Evolutionary changes in the genome coding for molecules of the Notch pathway from the simplest to the most complex organisms could have permitted the sprouting of distinct sister cell types and ensure their independent evolution by regulating cell-type specific traits. The *Notch* gene has been initially identified in *Drosophila melanogaster* [55]. Insects, *Ciona* species, sea urchin and amphioxus carry only one *Notch* copy [21, 22]. The two *Notch* copies in *Caenorhabditis elegans* (*C. elegans*), resulted from an independent duplication event within its linage [56], differ from the *Notch* copies from other taxa. Four *Notch* paralogues (i.e., *Notch1, Notch2, Notch3, Notch4*) have been found in invertebrates and vertebrates [9]. It is believed that *Notch1*, *Notch2* and *Notch3* have originated by two duplication events in vertebrates prior to the divergence of mammals, birds, reptiles, amphibians and teleost [21, 22] (Figs. 1 and 5). *Notch2* has emerged from *Notch1*, possibly at the first round of duplication events in vertebrates, whereas *Notch2* duplication led to the appearance of *Notch3*. The exclusive presence of a second *Notch1* copy in fishes might be due to an independent duplication event after the differentiation of tetrapoda and teleost fish [21, 57]. The *Notch4* gene has been identified only in mammals and its origin is still under debate [22]. *Notch* evolution in birds and reptiles is still unclear: *Notch3* has not yet identified in birds, while both *Notch3* and *Notch4* have not detected in reptiles [21, 22]*.* A more thorough sequencing of avian and reptile genomes could elucidate the *Notch* evolutionary gap between teleost fish and mammals. Albeit this lack of information, it is well-established that *Notch* genes are highly conserved throughout metazoans [21, 22]. There is still no evidence of the existence of *Notch* genes in any group besides metazoan phyla, suggesting that *Notch* appeared as a necessity for complex cellular communication and organization.

The canonical Notch signaling pathway mediates interactions between two neighboring cells, one of which is the signaling cell and the other is the receiving cell, via the physical interaction of the ligand with the Notch receptor at the cell surface [8]. It is well-established that the fine regulation of the Notch pathway is efficient for the activation of distinct downstream mechanisms in both developmental and evolution processes. Therefore, Notch is essential for the formation of complex and exquisite tissues that require often the cooperation of different cell types with discrete functions. By directing cell fates toward proliferation, differentiation, self-renewal, or cell death, Notch signaling is also involved in the assemblage of distinct cell populations that will accomplish the refined, ordinated and complex mechanisms for the generation of a unique tissue. For example, duplicated Notch paralogues expressed in the cerebral cortex resulted in progenitors’ clonal expansion and improved neurogenesis [58, 59]. Deletion of the partially duplicated *NOTCH* paralogues (*NOTCH2NL*) in the human cortex induced microcephaly, while their duplication caused megacephaly [58]. These findings suggest that appropriate Notch signaling supplementation in higher vertebrates might contribute to the evolution of specific tissues. The numerous and distinct roles of Notch signaling in vertebrates are facilitated by different combinations of ligands and receptors [60, 61], interactions through additional signaling molecules [62, 63] or addition of novel genes [58, 59]. These events determine the predominant role of Notch signaling in the evolution of tissues and organs [64–67].

The evolution of teeth could also depend on Notch signaling for the generation of new dental cell types from the already existing primitive dental cell types, thus allowing the formation of more complex dental structures such as the tooth enamel. Indeed, tooth morphology shows an astounding heterogeneity among vertebrates [68–73]. While all teeth display the same basic organization [74, 75], their positioning, shapes, and mineral composition vary considerably [68, 76–82]. Cartilaginous and bony fishes are characterized by either homodont or heterodont dentitions (i.e., no or little morphological variability within the same dentition) that are continuously renewed (polyphydonts) [70, 73, 74, 83–87]. The single teeth can have nevertheless highly complex morphologies, and their positioning and orientation within the jaw is thought to confer a certain level of functional specialization [88]. Reptiles and amphibians possess relatively simple teeth, which are often continuously replaced [72, 89, 90]. Mammals display more complex dental structures and generally exhibit a reduced tooth turnover [68, 89]. At the level of mineralization, the teeth of fishes are covered by enameloid, a highly mineralized hard tissue that contains collagenous and non-collagenous proteins [83, 91–95]. In contrast to fishes, teeth of reptiles, amphibians and mammals are covered by proper enamel [74]. Enamel does not contain collagenous proteins, and it is characterized by a higher degree of mineralization and a more complex structure when compared to enameloid of fishes [92]. Although tooth enamel in reptiles and amphibians is, with some exceptions [96], structurally simple and aprismatic [72, 74, 89, 97, 98], enamel in mammalian teeth is prismatic and characterized by the presence of organized bundles of hydroxyapatite crystals that confer it exceptional hardness and resistance to stresses [89, 99–101].

It was hypothesized that the reduction of tooth turnover in primordial mammals triggered the need for more durable teeth, leading to the formation of accurate and solid new enamel structures [89]. The complexity of enamel correlates with the specialization of the dental epithelium. In the mammalian dental epithelium, also called enamel organ, four distinct cell types have been identified based on histological analysis, gene expression analysis, functional characterization, and modern imaging techniques [102]. A similar organization of the dental epithelium was observed in other enamel-producing taxa, such as reptiles, where three to four dental epithelial layers were described [72, 103, 104]. In fishes, however, only two dental epithelial cell types are present [105].

**Fig. 6:**
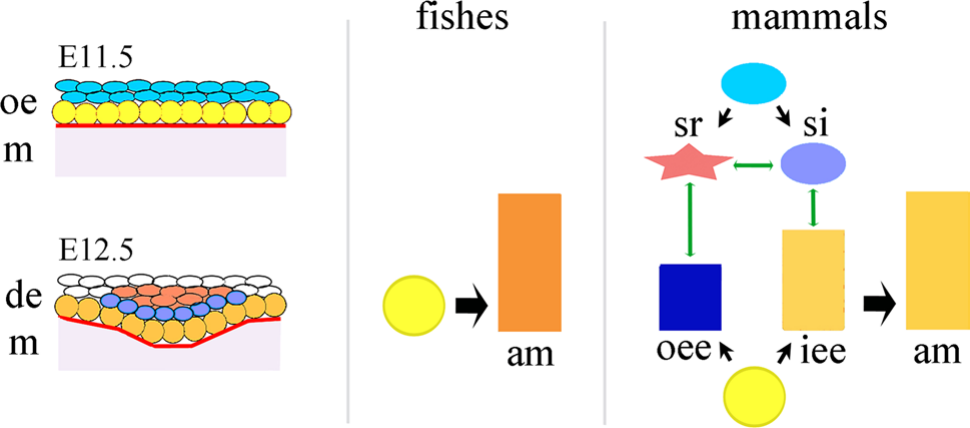
A model showing the generation of the enamel organ composed by different cell types in teeth. In fishes, only one specialized epithelial cell type, the ameloblast (am), can be distinguished in the tooth germ. In mice, oral epithelial (oe) cells in close contact with the mesenchyme (m; yellow color) give rise to two cell types, the inner enamel epithelial (iee) cells and outer enamel epithelial (ooe) cells, while the rest of the epithelial cells give rise to cells of the stratum intermedium (si) and stellate reticulum (sr). All these cell populations compose the enamel organ, which is an evolutionary unit essential for elaborating the extremely refined enamel structure in mammalian teeth. Physical interactions between all these cell types (green arrows) through the Notch signaling machinery are necessary for proper amelogenesis. In fishes, amelogenesis relies exclusively to ameloblasts (am), having as consequence the formation of a less elaborated structure called enameloid. *de* dental epithelium, *E* embryonic day

Previous studies in mammals have demonstrated that Notch signaling is essential for tooth development, morphology and tooth-specific mineral matrices deposition [36, 37, 39, 40, 42, 48, 51, 106]. Notch signaling defines the four dental epithelial cell lineages through the temporo-spatial differential expression pattern of the various Notch receptors and ligands during odontogenesis [41] (Fig. 2). However, it remains unclear how Notch signaling contributes to the establishment of distinct enamel or enamel-like structures in different species. Enamel formation represents a very sophisticated cellular process, as it requires a tightly controlled sequence of cell proliferation, differentiation, extracellular matrix secretion and re-absorbance, and crystal mineralization [100, 107]. This process needs to be tightly regulated spatially and temporally, as even minor changes can lead to functionally relevant alterations of the fine enamel structure [42, 101]. In mammals, all four dental epithelial cell types of the enamel organ are indispensable for the formation of a properly structured and mineralized enamel [42]. Among these, ameloblasts are the most characterized and directly responsible for the secretion and maturation of the enamel matrix [41, 100]. The role of the other three dental epithelial cell types (i.e., stratum intermedium, stellate reticulum and outer enamel epithelium) is not yet well-studied or understood. In lower vertebrates, such as different fish taxa (e.g., Teleosts), the enamel structure is less refined and organized than in mammals, which is indicative of a simpler and less precise mechanism for the formation of enamel. This procedure is carried out by a single epithelial cell type and requires mesenchymal-derived odontoblasts to co-participate in the processes of both organic matrices secretion and minerals deposition [74, 108]. Enameloid formation by a single cell type may represent a phylogenetically early stage in the differentiated capability of the evolving ameloblasts [105, 109]. We can assume that a primitive dental epithelial cell type, forming a set of cells within the enamel organ, has changed during evolution and gave rise to additional, closely related cell types. It is indeed well accepted that the number of cell types has changed during animal evolution [110]. Basal metazoans have relatively few cell types, indicating that there was a large expansion of cell type diversity before the bilaterian ancestor [111]. This increase of cell types was accompanied by the shift from few, multifunctional cells, towards multiple, specialized sister cells. These new cells can exert precise functions previously performed by a primitive single cell, or acquire completely new functions [112]. In many cases, this segregation and divergence is driven by gene duplication [112], by expression of novel genes, or by co-option of already existing genes for new cellular functions [110, 112]. By these means, sister cells can synergistically lead to the formation of extremely complex tissues that could not be generated by single multifunctional cells. We propose that the Notch signaling pathway, and in particular the differential expression of its ligands and receptors, could be a key determinant of cell specification and functional segregation in the evolution of teeth, and most probably in other organs and tissues (Fig. 6). Notably, Notch could also exert its biological functions via non-canonical signaling, as it does during neurogenesis and myogenesis [113]. However, there is no yet evidence of involvement of the non-canonical Notch signaling during odontogenesis and amelogenesis.

**Fig. 7:**
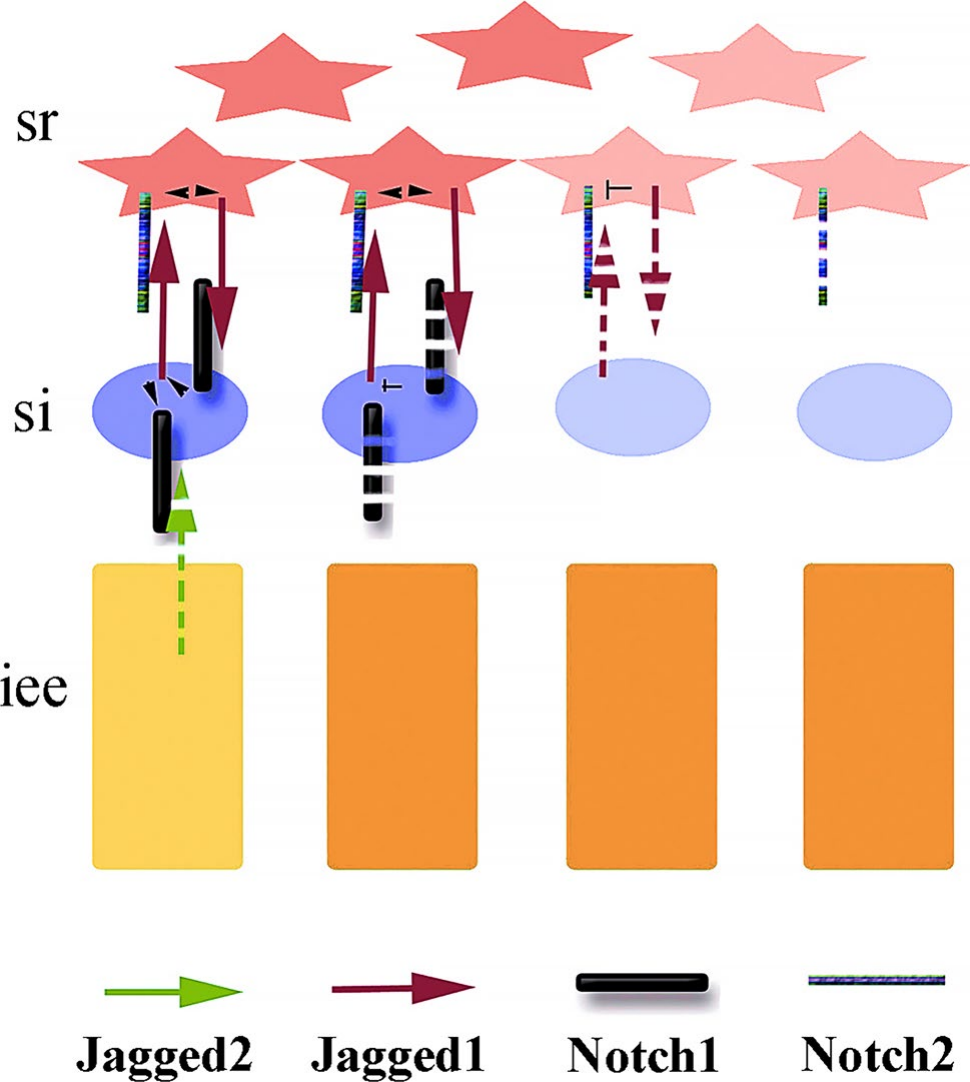
Hypothetical model of Notch signaling action in the successive cell layers of the enamel organ during mouse odontogenesis. The defined expression pattern of the Notch ligands Jagged1 and Jagged2 (arrows) and the Notch receptors Notch1 and Notch2 (bars) in different cell types of the enamel organ with discrete and complementary functions contribute to the formation of the highly refined and well-structured mammalian enamel. Deletion of Jagged2 (green spaced dotted arrow) in inner enamel epithelium (iee) cells results in Notch1 down-regulation (thin spaced dotted bar) in stratum intermedium (si) cells, followed by Jagged1 (dark red spaced dotted arrows) down-regulation in stratum intermedium and of Jagged1 and Notch2 (thin spaced dotted bar) in stellate reticulum (sr) cells, according to the Notch specific lateral inhibition mode of action. Loss of interactions between Notch and Jagged proteins may either shift the behavior of these cells or initiate loss of their identity, thus returning back the evolutionary path by impairing the specialization of the cells that contribute to mammalian amelogenesis. As a consequence, amelogenesis will be carried out by only one single cell type, thus generating structures resembling more enameloid of fishes than enamel of mammals

Studies in fishes have shown that members of the Notch signaling pathway are actually expressed during tooth development [105]. In cichlid fishes, *Notch1* and *Jagged2* expression are associated with the successional lamina (i.e., the structure responsible for tooth renewal, and hence new tooth buds), while during the maturation and secretion stages they are co-expressed in ameloblasts and the adjacent epithelial cells [105]. It is noteworthy that in the teeth of fishes the expression domains of *Notch1* and *Jagged2* are largely overlapping [105], and they are thus not obviously distinct and demarcated as in the teeth of mice, where the expression of Notch ligands and receptors clearly defines the four cell types of the enamel organ (Fig. 2F) [41]. No studies described the expression of Notch ligands and receptors in other taxa such as reptiles or amphibians. In mice, mutations or inhibition of Notch signaling affects teeth and most specifically the formation and structure of enamel [36, 37, 42, 48, 51]. Constitutive deletion of *Jagged2* is perinatally lethal in mice, and affects dental epithelial progenitor cells ability to form ameloblasts, leading to the development of teeth with abnormal morphology and lacking enamel [51]. Previous studies have shown that the postnatal inhibition of Notch signaling leads to alterations in cell–cell contacts at the ameloblasts-stratum intermedium interface, without major direct effects on ameloblasts [36]. Nevertheless, this disturbance eventually results in enamel defects [36]. However, we have shown recently that the epithelial deletion of Adam10, a membrane-bound metalloproteinase regulating Notch signaling, causes the loss of the stratum intermedium layer and the disorganization of ameloblasts that triggers deficient enamel formation [42]. Furthermore, deletion of the Jagged1 ligand in dental epithelium dysregulates the expression of genes involved in the Notch pathway (e.g., *Notch1*, *Notch2*, *Hes5*), as well as of enamel-specific genes (e.g., *Amelx*, *Enam*) [48]. Moreover, deletion of *Jagged1* in the dental epithelium of transgenic mice leads to tooth crown shape modifications convergent to those observed along Muridae evolution [48]. Analogous mechanisms have been observed in humans. Mutations in *TSPEAR* lead to enamel defects via down-regulation of NOTCH signaling in human patients [114]. Similarly, mutations in *AMELX*, which cause severe enamel defects in humans, are associated with aberrant overexpression of *NOTCH1* in ameloblasts [115]. Therefore, Notch signaling deregulation within the enamel organ, which can be seen as an evolutionary unit, do not allow sister cell lines to express distinct molecular programs that maintain their cellular specificity, resulting in defective enamel formation (Fig. 7) [42]. In fishes, pharmacological inhibition of Notch signaling impairs tooth renewal [105], while to date no studies investigated its roles in fish dental epithelium differentiation and enameloid formation. Molecules of the Notch pathway control the dental cell-type specificity and mediate their distinct responses to common signals [48]. On a broader scale, Notch signaling is the central hub of a molecular network that determines cell fate choice throughout animal development, homeostasis, and regeneration via lateral inhibition [8, 18, 116–119]. A flat hierarchy of gene regulation [18, 118] upon Notch signaling deletion could thus revert the evolutionary path, impairing the specialization of the cells that contribute to amelogenesis and thus generating structures resembling more enameloid of fishes than enamel of mammals [42]. Hence, loss of interactions between Notch and Jagged/Delta-like proteins within the enamel organ may either shift the behavior of cell types or initiate the suppression of complementary dental epithelial cell fates.

The expansion of the functions of the Notch signaling pathway in the generation of highly specialized cell types could be due not only to the duplications of the genes coding for its ligands and receptors, but also to the refinement of their expression domains. Many *loci* involved in the patterning and growth of the musculoskeletal system and dental apparatus in vertebrates are controlled by complex *cis*-regulatory systems, as these systems permit highly compartmentalized and fine-tuned control of gene expression in specific cellular and tissue-specific contexts [120–128]. Indeed, it is becoming clear that changes in gene expression patterns play a pivotal role in the evolution of complex morphological traits [48, 129–131]. These changes are more often due to mutations in cis-regulatory sequences, rather than coding sequences, the latter of which can pleiotropically alter the expression domains of key signaling molecules [129]. Members of the Notch pathway should also be subject to this type of fine-tuned tissue-specific control and future functional genomics study on developing teeth and their cell populations will likely reveal this to be the case. These leads us to the suggestion that the concomitant duplication of Notch ligands and receptors, and their progressively more defined expression domains via the evolution of associated complex *cis*-regulatory systems could be the driving force of the generation of highly specialized cell types during the evolution of teeth [129]. The proposed correlation between Notch receptors and ligands, and the generation and maintenance of distinct dental cell types, could represent a general mechanism underlying the evolution of specialized cell types in metazoa.

## Data Availability

This is not applicable to the present article.
